# Predicting vessel recanalization in extracranial internal carotid artery dissection: a nomogram based on ultrasonography and clinical features

**DOI:** 10.3389/fneur.2025.1498182

**Published:** 2025-04-07

**Authors:** Xinchun Xu, Yanhong Yan, Yafeng Qu, Lianlian Zhang, Pinjing Hui

**Affiliations:** ^1^Department of Neurosurgery, The First Affiliated Hospital of Soochow University, Suzhou, China; ^2^Department of Stroke Center, The First Affiliated Hospital of Soochow University, Suzhou, China; ^3^Department of Ultrasound, The Affiliated Zhangjiagang Hospital of Soochow University, Suzhou, China

**Keywords:** extracranial internal carotid artery dissection, vessel recanalization, carotid duplex ultrasound, predictive model, nomogram

## Abstract

**Background:**

Extracranial internal carotid artery dissection (EICAD) is a prominent factor in ischemic stroke in young patients, and vessel recanalization is correlated with stroke recurrence. We propose to determine the possible association between carotid duplex ultrasound (CDU) features, clinical factors, and vessel recanalization in EICAD patients.

**Methods:**

In the current retrospective study, data from 202 patients diagnosed with EICAD by CDU and confirmed by computed tomography angiography (CTA) or high-resolution magnetic resonance imaging (HRMRI) were encompassed. Patients were randomized 7:3 into training cohort (*n* = 142) and validation cohort (*n* = 60). The least absolute shrinkage and selection operator (LASSO) regression analysis and multivariate logistic regression analysis were used to build a nomogram to predict recanalization. At last, we assessed the performance of the nomogram with an area under the receiver operating characteristic curve (AUC), calibration curve, decision curve analysis (DCA), and clinical impact curve (CIC).

**Results:**

The nomogram included CDU features (intramural hematoma, Intraluminal thrombus, and stenosis degree) and age, with AUC values of 0.906 (95% CI: 0.857–0.946) and 0.903 (95% CI: 0.820–0.963) in the training cohort and the validation cohort, respectively. Using a probability cutoff of 0.5 derived from the Youden index, patients were stratified into high-risk (recanalization probability <50%) and low-risk groups (≥50%). DCA showed that the nomogram performed significantly better across various threshold probabilities, and CIC demonstrated that the nomogram offers superior net benefit across a broad range of threshold probabilities, indicating its significant predictive value.

**Conclusion:**

A nomogram depended on CDU and clinical features could accurately predict recanalization in EICAD patients. The nomogram may facilitate early identification of high-risk patients and personalized therapeutic strategies.

## Introduction

Extracranial internal carotid artery dissection (EICAD) is a disorder characterized by the passage of blood via a rip in the arterial wall layers, resulting in the blood entering the space between these layers, causing the carotid wall to separate into two layers and interfering with blood flow, which can lead to secondary stenosis or aneurysmal dilatation (1). Carotid artery dissection (CAD) accounts for around 25% of strokes in young individuals, making it a significant factor in stroke occurrence among individuals in their youth and middle age (2). Therefore, the accurate diagnosis and effective treatment of carotid artery dissection, as well as the enhancement of patients’ prognosis, are of crucial in clinical practice. Digital subtraction angiography (DSA) has traditionally been considered the most reliable method for diagnosing EICAD. However, this technique is an invasive examination and cannot clearly show the morphology of arterial wall, so it has certain limitations in the clinical diagnosis and treatment process (3). In recent times, high-resolution magnetic resonance imaging (HRMRI) has been increasingly employed in clinical practice. It has a high detection rate for intramural hematoma and can clearly show the structure of the vessel wall. However, it is time-consuming (4). Carotid Doppler ultrasound (CDU) has emerged as a valuable diagnostic modality for the evaluation of EICAD. It offers several advantages over other imaging techniques, such as being non-invasive and cost-effective, and it can observe the lumen and artery wall of the extracranial internal carotid artery in real-time. Therefore, CDU is of great value in evaluating the variations in vascular wall structure of EICAD patients.

Previous studies have been conducted on the recanalization rate of CAD (5–7), while limited attention has been given to the influence factors of recanalization. Furthermore, the nomogram is progressively employed as a visual aid for the purpose of illness prevention. However, few studies have combined CDU characteristics and clinical factors to establish a nomogram to evaluate the recanalization of EICAD. We aim to combine CDU features and clinical factors to identify those factors that are significantly correlated to vessel recanalization and to establish a nomogram to forecast the recanalization probability.

## Materials and methods

### Patient data

**Figure 1:**
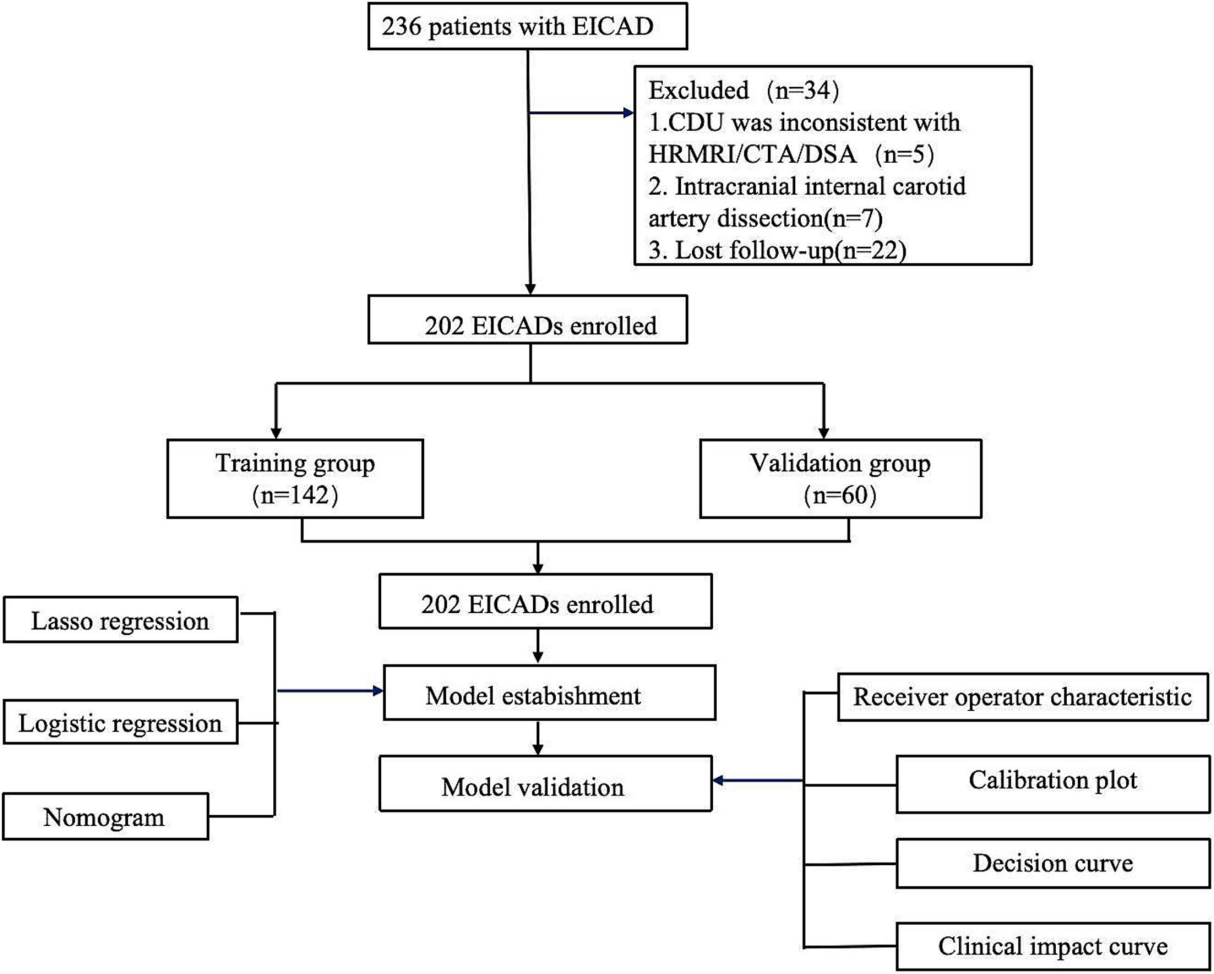
Flow diagram of the study participants.

This study was approved by the Ethics Committee of the first affiliated hospital of Soochow University (No. 2021196). The individuals provided informed consent before enrolling in the study. The clinical and imaging data of EICAD patients were admitted from August 1, 2015, to June 30, 2023. The inclusion criteria were: (1) patients with suspected EICAD who received CDU examinations and were confirmed by following HRMRI and/or CTA; (2) patients with available follow-up ultrasound examinations for assessment. Clinical suspicion of EICAD depended on one or more of the following signs: neck pain, headache, and Horner’s syndrome. Patients were excluded if: (1) patients had bilateral or multiple-segment CAD; (2) patients had incomplete medical records or missing data necessary for analysis; and (3) patients had contraindications to anticoagulant or antiplatelet therapy. In our research, a total of 202 individuals with EICAD were included. All patients received anticoagulant therapy (warfarin, dabigatran, or rivaroxaban, maintained at an international normalized ratio [INR] of 2.0–3.0) or antiplatelet therapy (aspirin 100 mg/day and/or clopidogrel 75 mg/day) upon initial diagnosis. The choice of therapy was guided by protocols, considering factors such as dissection severity and bleeding risk (8). Subsequently, the patients who had joined were allocated into two groups, namely a training group (*n* = 142) and a validation group (*n* = 60), using a randomization process with a ratio of 7:3. Figure 1 displays the specifics of the research design.

### Data collection

The following demographic and clinical data were obtained: age, gender, neck pain, headache, Horner’s syndrome, hypertension, diabetes, smoking, neck trauma, prior stroke, low-density lipoprotein cholesterol (LDL-C, mmol/L), High-density lipoprotein cholesterol (HDL-C, mmol/L), Total cholesterol (TC, mmol/L), triglycerides (mmol/L), glycated hemoglobin (HbA1c), homocysteine (HCY, umol/L), high-sensitivity C reactive protein (hs-CRP, mg/L), the National Institutes of Health Stroke Scale (NIHSS), medical treatment (antiplatelets, anticoagulant, or treatment with both agents), and initial and follow-up imaging.

### Imaging examinations

CDU imaging was conducted employing an iU-Elite scanner (Phillips Medical System, Bothell, Washington) that has a high-frequency L9-3 probe. All examinations were performed by a 10-year experienced sonographer who specialized in vascular ultrasound. During the examination, the patient was positioned in a supine posture with his head rotated toward the opposite carotid artery. Two-dimensional real-time imaging was performed to scan the transverse section first and then the longitudinal section.

The CTA examination used a 320-row-detector CT scanner (Aquilion ONE, Toshiba Medical Systems, Tokyo, Japan) with a high-spaced spiral scan mode. The imaging parameters were as follows: 100 kV tube voltage, 1.0 spacing, 3 mm slice thickness, 3 mm gap, rotation time of 0.5 s, and scanning time of 3–4 s, delay time of 2 s. After routine scanning, patients received 40 mL of intravenous iodine contrast agent (Isovue-M®, United States) at 4–5 mL/s, followed by 50 mL of saline at the same injection rate. All images were transmitted to the workstation for post-processing of multiplanar reconstruction (MPR), maximum density projection (MIP), surface reconstruction (CPR), and volume reorganization (VR).

The patients received HRMRI utilizing a 3-Tesla MRI system (Ingenia, Philips Healthcare, Best, the Netherlands) equipped with an eight-channel integrated head and neck coil. The imaging procedure encompassed T1W black-blood (BB) sequence, 3D time-of-flight (TOF) MRA, fat-suppressed (FS) T2-weighted BB imaging, and proton-density-weighted volume isotropic turbo-spin-echo acquisition (PD-VISTA) sequences. The T1W-BB and T2W-BB sequences were obtained in the axial orientation. The PD-VISTA pictures were obtained in the coronal orientation. The following variables were used: 3D TOF MRA with a repetition time (TR) of 15 ms and an echo time (TE) of 3.5 ms, the field of view (FOV) was 220 × 128 × 148 mm^3^, with a matrix size of 512 × 512, and the total scanning time was 3.5 min; PD-VISTA: TR of 34 ms and TE of 2,000 ms, the FOV was 189 × 162 × 40 mm^3^, with a matrix size of 480 × 480, and the total scanning time was 2 min 40 s. T1W-BB has a scanning time of 4 min 58 s with a TR/TE of 20 ms/968 ms, FOV of 100 × 100 × 17 mm^3^, and a matrix size of 352 × 352; FS-T2W-BB has a scanning time of 4 min 13 s with a TR/TE of 2,432 ms/40 ms, FOV of 100 × 100 × 15 mm^3^, and a matrix size of 384 × 384.

### Image analysis

Two experienced radiologists, each with over five years of diagnostic experience, reviewed baseline and follow-up imaging independently using PACS workstation. On CDU and HRMRI, the presence of EICAD was defined if images had either (9–12): (1) double lumen; (2) intimal flap; (3) intramural hematoma; (4) intraluminal thrombus; or (5) a tapering stenosis with distal occlusion with no atherosclerotic disease exists. On CTA, the presence of EICAD was defined as one of these features: (1) intimal flap; (2) an enlargement of the dissection artery; (3) dissecting aneurysm; or the lumen is small and irregularly formed, with a thrombus forming a crescent shape around it. There is also a thin ring of enhanced tissue around the thrombus (13, 14). Stenoses of the EICA were defined based on guidelines by North American Symptomatic Carotid Endarterectomy Trial (NASCET) (15). The degree of stenosis was categorized based on the following ranges: mild (<50%), moderate (50–69%), severe (70–99%), and complete occlusion.

### Outcome definition

**Figure 2:**
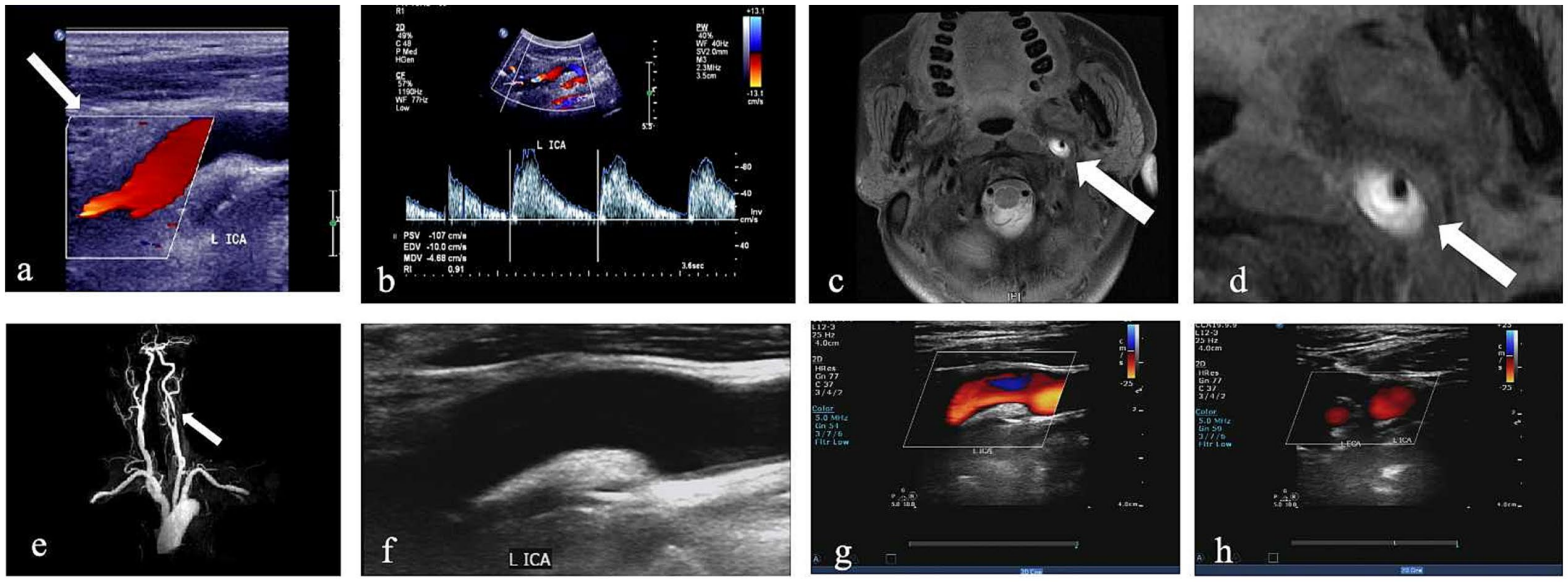
CDU imaging for one patient with left internal CAD. **(a,b)** Greyscale and pulsed-wave Doppler with hypoechogenic intramural hematoma (white arrow) and lumen narrowing. **(c,d)** Corresponding HRMRI with evidence of a crescent-shape hyperintense on axial FS-T2W images and enlarged view of FS-T2W (white arrow). **(e)** 3D time-of-flight (TOF) magnetic resonance angiography imaging. **(f–h)** CDU done 6 months later shows recanalization after treatment; the wall structure of the ICA returned to normal, and the blood flow signal was completely filled.

Recanalization was defined as the reopening of the injured vessels with no vascular abnormalities, and blood flow velocity and spectral morphology were normal, which was assessed using follow-up CDU (Figure 2). Patients underwent initial CDU assessment at 1 month post-diagnosis, followed by serial evaluations every 3 months until recanalization was confirmed or the 1-year endpoint was reached.

### Statistical analysis

The statistical analyses were conducted employing the R program (version 4.2.2; R Foundation for Statistical Computing, Vienna, Austria) and its related data packages. The dataset was partitioned into training and validation cohorts employing a random allocation ratio of 7:3, and the variables were then examined. The continuous data were reported as the mean and standard deviation (SD) or the median (interquartile range [IQR]), whereas the categorical data were reported as numbers expressed as a percentage (%). The categorical variables were analyzed employing either the Pearson chi-square test or Fisher’s exact test, while the continuous variables were examined employing either the Student’s t-test or Mann–Whitney U test. The training cohort deployed the least absolute shrinkage and selection operator (LASSO) logistic regression analysis to identify the most suitable variables for recanalization. Subsequently, a multivariate logistic regression analysis (MVLR) was conducted by including the chosen characteristics in the LASSO regression model. The outcomes of the MVLR were used to create the ultimate forecasting model. Subsequently, we constructed a prognostic nomogram to estimate the likelihood of recanalization. The nomogram is internally verified using the training set and externally validated using the validation set. The discriminating performance of the model was determined by employing the receiver operating characteristic (ROC) curve. A calibration plot was employed to determine the correlation between the reported risk and the estimated risk. Furthermore, a decision curve analysis (DCA) was conducted to ascertain the net benefit threshold of the prediction, and clinical impact curve (CIC) were generated to assess the clinical utility and net benefits of the model exhibiting the best diagnostic accuracy. AUC values were compared using the Delong test. The total points derived from the nomogram were translated into predicted probabilities of recanalization. Based on the Youden index calculated from the ROC curve analysis, a cutoff value of 0.5 (50% probability) was selected to stratify patients into high-risk (probability<50%) and low-risk (probability ≥50%) groups for recanalization. Significance was attributed to variations when the *p*-value was <0.05.

## Results

### Patient characteristics

The study included 202 patients with EICAD, of whom 90 (44.6%) achieved recanalization. The mean age of the cohort was 48.6 ± 10.9 years, with a male predominance (76.2%). Key clinical characteristics included hypertension (46.5%), diabetes (14.3%), and smoking history (31.2%). Among imaging features, intramural hematoma (IMH) was present in 62.9% of patients, intraluminal thrombus in 27.2%, and complete occlusion in 46.5%. Table 1 provides a summary of the baseline characteristics of the research population.

**Table 1:** Clinical characteristics of EICAD patients.

Characteristic	Recanalization			*p*-value
	Overall, *N* = 202	yes, *N* = 90	no, *N* = 112	
Age	48.6 ± 10.9	43.1 ± 10.3	53.1 ± 9.2	<0.001
Gender				0.062
Female	48 (23.8)	27 (30.0)	21 (18.8)	
Male	154 (76.2)	63 (70.0)	91 (81.2)	
Neck pain	23 (11.4)	11 (10.0)	12 (13.3)	0.435
Headache	29 (14.4)	17 (15.2)	12 (13.3)	0.710
Hornor sign	16 (7.9)	9 (8.0)	7 (7.7)	0.946
Hypertension	94 (46.5)	41 (45.6)	53 (47.3)	0.803
Diabetes	29 (14.3)	13 (11.6)	16 (17.8)	0.214
Smoking	63 (31.2)	15 (16.7)	48 (42.9)	<0.001
Neck trauma	32 (15.8)	17 (15.2)	15 (16.7)	0.773
Prior stroke	18 (8.9)	4 (4.4)	14 (12.5)	0.046
NIHSS score	3.0 (0.0, 7.8)	4.0 (0.0, 7.5)	3.0 (0.0, 7.3)	0.934
Laboratory examination				
TC (mmol/L)	3.83 (3.22, 4.65)	3.85 (3.31, 4.65)	3.83 (2.97, 4.63)	0.245
HDL-C (mmol/L)	1.05 (0.86, 1.24)	1.04 (0.91, 1.16)	1.05 (0.81, 1.27)	0.352
LDL-C (mmol/L)	2.22 (1.65, 3.00)	2.22 (1.89, 2.95)	2.20 (1.54, 3.00)	0.309
Triglycerides (mmol/L)	9.8 (6.5,13.6)	9.6 (7.1,13.1)	10.1 (7.4,13.8)	0.412
HbA1c (%)	5.9 (5.5, 6.5)	5.6 (3.89, 6.4)	6.2 (4.1, 6.7)	0.966
HCY (umol/L)	9.4 (8.0, 11.6)	9.2 (7.7, 11.6)	9.7 (8.2, 11.2)	0.309
hs-CRP (mg/L)	2.6 (1.0, 6.3)	2.4 (0.8, 4.6)	4.0 (1.2, 9.2)	0.101
NIHSS score	3.0 (0.0, 7.8)	4.0 (0.0, 7.5)	3.0 (0.0, 7.3)	0.934
Medical treatment				
Antiplatelets	73 (36.1)	39 (43.3)	34 (30.4)	0.056
Anticoagulant	35 (17.3)	24 (21.4)	11 (12.2)	0.886
Treated with both agents	96 (47.5)	32 (35.6)	64 (57.1)	0.002
Carotid dissection imaging				
Double lumen	25 (12.4)	17 (15.2)	8 (8.9)	0.177
Intimal flap	29 (14.4)	17 (15.2)	12 (13.3)	0.710
IMH	127 (62.9)	37 (41.1)	90 (80.4)	<0.001
Intraluminal thrombus	55 (27.2)	32 (35.6)	23 (20.5)	0.017
Stenosis degree				<0.001
Mild	30 (14.9)	21 (18.8)	9 (10.0)	
Severe	40 (19.8)	25 (22.3)	15 (16.7)	
Moderate	38 (18.9)	29 (25.9)	9 (10.0)	
Occlusion	94 (46.5)	36 (33.0)	58 (64.4)	

Values are presented as mean (SD), median (25th-75th percentile), or number of patients (%). Welch Two Sample t-test; Pearson’s Chi-squared test; Fisher’s exact test; Wilcoxon rank sum test. EICAD, extracranial carotid artery dissection; TC, Total cholesterol; HDL-C, High-density lipoprotein cholesterol; LDL-C, Low-density Lipoprotein cholesterol; HbA1c, Glycated hemoglobin; HCY, homocysteine; hs-CRP, high sensitivity C reactive protein; NIHSS, National Institutes of Health Stroke Scale; IMH, intramural hematoma; IQR, interquartile range.

The patients were allocated into training and validation cohorts employing a random selection process. The training cohort included 142 individuals, while the validation cohort consisted of 60 patients at a ratio of 7:3 and summarized their CDU and clinical characteristics (Table 2). Overall, the baseline characteristics of the study population were similar between the training cohort and the validation cohort, indicating that they can be considered comparable for further analysis.

**Table 2:** Patient demographics and baseline characteristics.

Characteristic	Training Cohort *N* = 142	Validation Cohort *N* = 60	*p*-value
Age	48.2 ± 11.3	51.4 ± 10.1	0.212
Gender			0.926
Female	34 (23.9)	14 (23.3)	
Male	108 (76.1)	46 (76.7)	
Neck pain	16 (11.3)	7 (11.7)	0.823
Headache	21 (14.8)	8 (13.3)	0.756
Horner sign	10 (7.0)	6 (10.0)	0.573
Hypertention	64 (45.1)	30 (50.0)	0.620
Diabetes	20 (14.1)	9 (15.0)	0.685
Smoking	45 (31.7)	18 (30.0)	0.813
Neck trauma	23 (16.2)	9 (15.0)	0.689
Prior stroke	14 (9.8)	4 (6.7)	0.467
NIHSS score	3.0 (0.0, 7.0)	4.0 (1.0, 8.0)	0.558
Laboratory examination			
TC (mmol/L)	3.78 (3.16, 4.65)	3.98 (3.40, 4.80)	0.315
HDL-C (mmol/L)	1.05 (0.86, 1.21)	1.03 (0.89, 1.24)	0.803
LDL-C (mmol/L)	2.15 (1.65, 2.98)	2.29 (1.84, 3.01)	0.554
Triglycerides (mmol/L)	9.6 (7.3,12.7)	9.8 (7.6,13.1)	0.375
HbA1c (%)	5.90 (5.50, 6.90)	5.70 (5.40, 6.30)	0.181
HCY (umol/L)	9.4 (8.0, 11.6)	9.4 (7.4, 11.6)	0.730
hs-CRP (mg/L)	2.7 (1.2, 6.3)	2.5 (0.7, 6.4)	0.359
Medical treatment			
Antiplatelets	57 (40.1)	16 (26.7)	0.069
Anticoagulant	27 (19.0)	6 (10.0)	0.100
Treated with both agents	58 (40.8)	33 (55.0)	0.061
Carotid dissection imaging			
Double lumen	18 (12.7)	7 (11.7)	0.555
Intimal flap	19 (13.4)	10 (16.7)	0.587
IMH	92 (64.8)	35 (58.3)	0.386
Intraluminal thrombus	39 (27.5)	16 (26.7)	0.907
Stenosis degree			0.271
Mild	18 (12.7)	12 (20.0)	
Moderate	27 (19.0)	13 (21.7)	
Severe	25 (17.6)	13 (21.7)	
Occlusion	67 (47.2)	27 (45.0)	

Values are presented as mean (SD), median (25th-75th percentile), or number of patients (%). Welch Two Sample t-test; Pearson’s Chi-squared test; Fisher’s exact test; Wilcoxon rank sum test. TC, Total cholesterol; HDL-C, High-density lipoprotein cholesterol; LDL-C, Low-density Lipoprotein cholesterol; HbA1c, Glycated hemoglobin; HCY, homocysteine; hs-CRP, high sensitivity C reactive protein; NIHSS, National Institutes of Health Stroke Scale; IMH, intramural hematoma; IQR, interquartile range.

### Clinical outcomes during follow-up

During follow-up, 16 out of 202 patients (7.9%) experienced cerebrovascular events. These included 12 ischemic strokes, and 4 transient ischemic attacks (TIA). Patients without recanalization had a significantly higher incidence of cerebrovascular events (13/112, 11.6%) compared to those with recanalization (3/90, 3.3%; *p* = 0.03). Most events occurred within the first 6 months post-diagnosis (14/16, 87.5%), aligning with the period of highest thrombotic risk in EICAD.

### Predictive model

**Figure 3:**
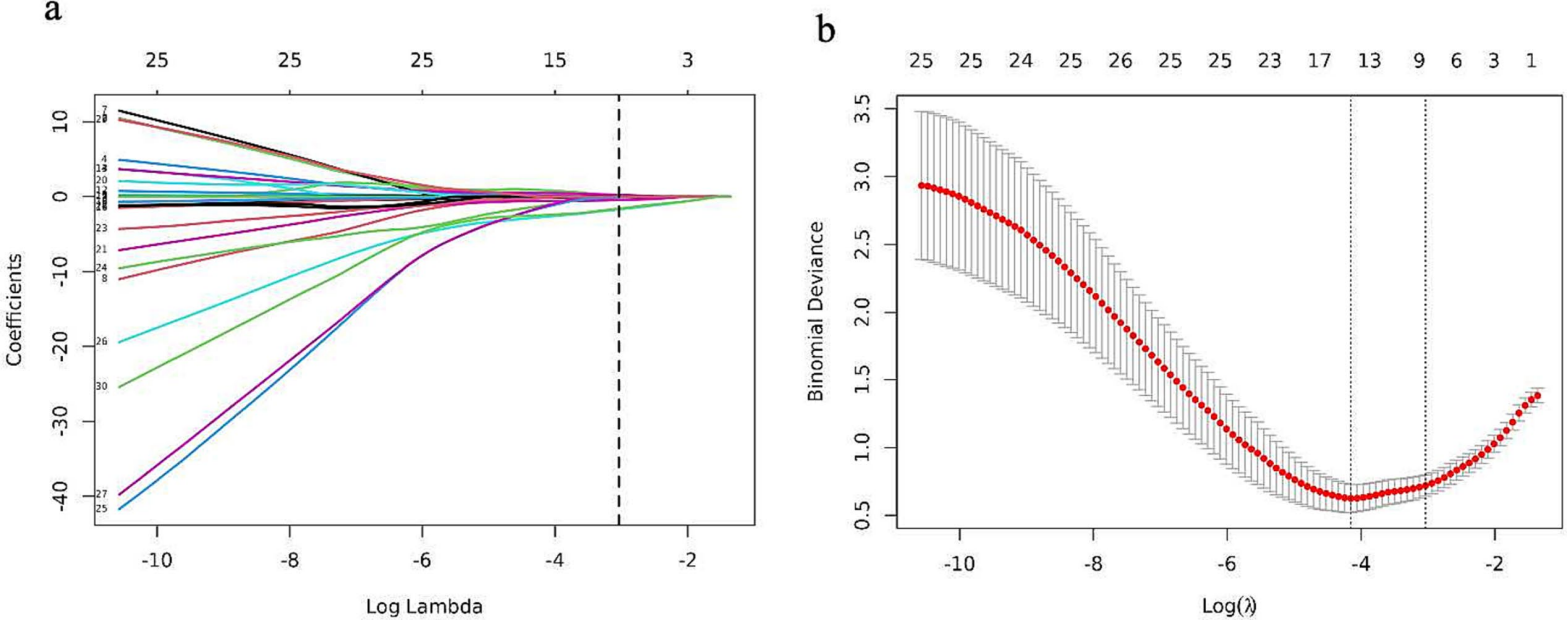
Features selection by the LASSO binary logistic regression model. **(a)** A visualization of the coefficient profile is generated using log (lambda) values. **(b)** The penalty value that maximizes performance has been determined using a 10-fold cross-validated (CV), and seven features have been picked based on their CV error, which is within one standard error of the minimum.

The candidate variables shown in Table 2 were initially included in the original model. Subsequently, LASSO regression analysis was conducted on the training cohort, resulting in the selection of seven possible predictors: age, smoking, LDL-C, treated with antiplatelets and anticoagulant, IMH, Intraluminal.thrombus, and stenosis degree. The coefficients are provided in Table 3, while a graphical representation of the coefficient profile may be seen in Figure 3a. Figure 3b displays a cross-validated (CV) error curve of the LASSO regression model. The model that had the most regularized and parsimonious approach and had a CV error that was within one standard error of the minimum consisted of 7 variables.

**Table 3:** The coefficients of LASSO regression analysis.

Coefficient	Variable
5.41624887	(Intercept)
−0.08715714	Age_level_
0.00000000	gender_level_male
0.00000000	neck pain_level_yes
0.00000000	headache_level_yes
0.00000000	Hornor sign_level_yes
0.00000000	hypertension_level_yes
0.00000000	diabetes_level_yes
−0.03472626	smoking_level_yes
0.00000000	Neck trauma_level_yes
0.00000000	prior.stroke_level_yes
0.00000000	TC._level_
0.00000000	HDL-C_level_
0.22847481	LDL-C_level_
0.00000000	HbA1c…_level_
0.00000000	HCY._level_
0.00000000	hs-CRP_level_
0.00000000	NIHSS_level_
0.00000000	Antiplatelets_level_yes
0.00000000	Anticoagulant_level_yes
−0.45366172	Treated.with.both.agents_level_yes
0.00000000	Double.lumen_level_yes
0.00000000	Intimal.flap_level_yes
−0.11460475	IMH_level_yes
−1.71225982	Intraluminal.thrombus_level_yes
0.00000000	Stenosis degree_level_Moderate
0.00000000	Stenosis degree_level_Severe
−1.59566650	Stenosis degree_level_Occlusion

MVLR analysis of these seven variables is shown in Table 4. The findings of the multivariate regression analysis indicated age (*p* < 0.001), IMH (*p* < 0.001), and occlusion (*p* = 0.001) as vessel recanalization risk factors.

**Table 4:** Results of the multivariate logistic analysis based on the training cohort.

Variables	β	S.E	Z	*p*	OR (95%CI)
Age	−0.170	0.038	4.505	<0.001	0.844 (0.784,0.909)
Smoking	−0.744	0.610	1.219	0.223	0.475 (0.144,1.572)
IMH	−5.532	1.419	3.898	<0.001	0.645 (0.161,2.589)
Intraluminal thrombus	−2.556	1.324	1.930	0.054	0.078 (0.006,1.040)
Stenosis degree					
Mild					Ref
Moderate	−1.380	0.908	1.519	0.787	0.252 (0.042,1.493)
Severe	−0.439	0.709	0.619	0.536	0.645 (0.161,2.589)
Occlusion	−2.747	0.819	3.352	0.001	0.064 (0.013,0.320)

IMH, intramural hematoma; OR, odds ratio; CI, confidence interval.

### Nomogram establishment and validation

**Figure 4:**
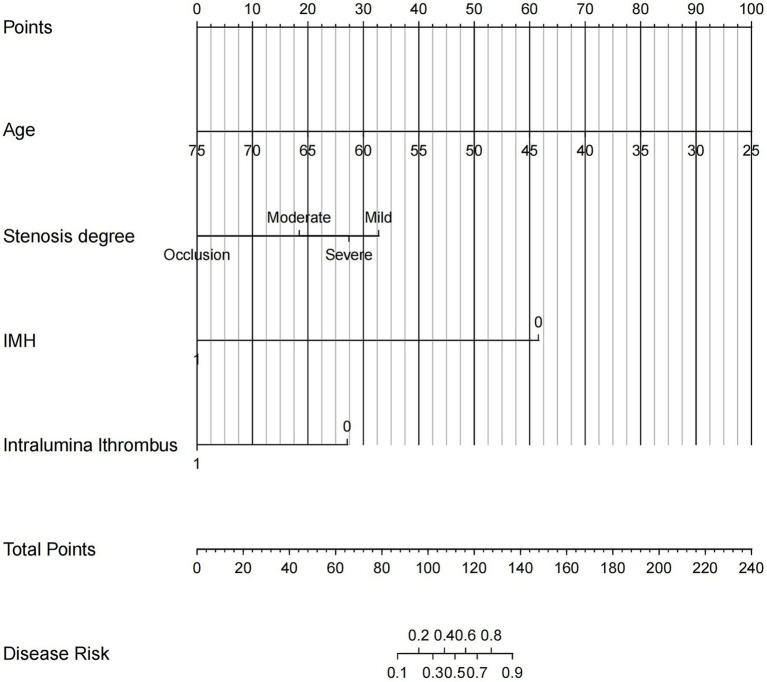
The nomogram based on CDU and clinical features was established for predicting vessel recanalization in patients with EICAD. Each patient’s value is assessed using several scoring systems, and the total scores are plotted on the total points axis to determine the likelihood of vessel recanalization. IMH, intramural hematoma.

The final logistic model encompassed four autonomous variables (age, IMH, Intraluminal.thrombus, and stenosis degree) were formulated as a user-friendly nomogram (Figure 4).

**Figure 5:**
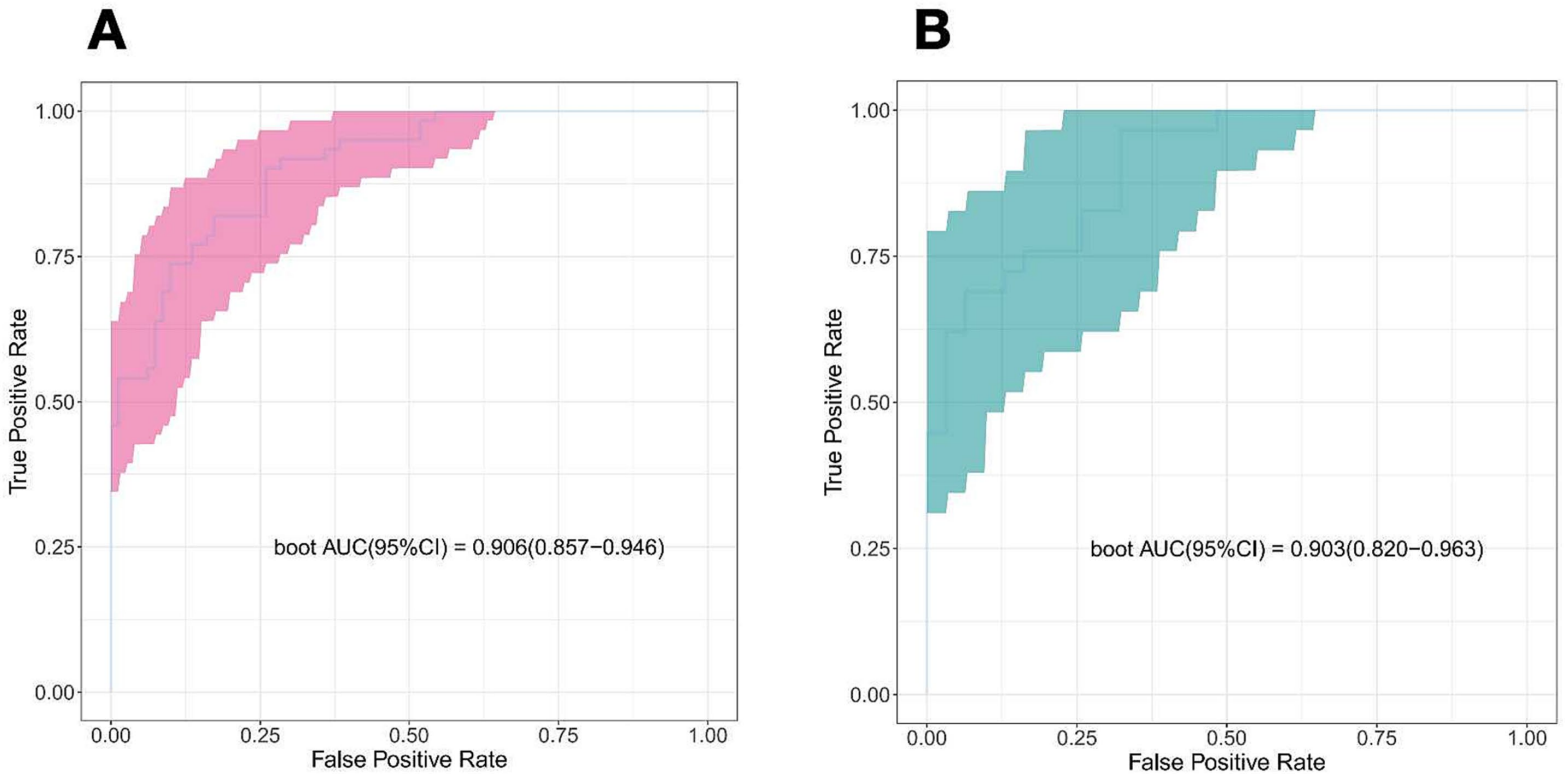
Receiver operator characteristic curve of the nomogram prediction model. **(A)** Train cohort. **(B)** Validation cohort.

The discriminating power of the prediction model was assessed using the area under the curve (AUC). The AUC was found to be 0.906 in the training cohorts and 0.903 in the validation cohorts, indicating outstanding performance (Figure 5).

**Figure 6:**
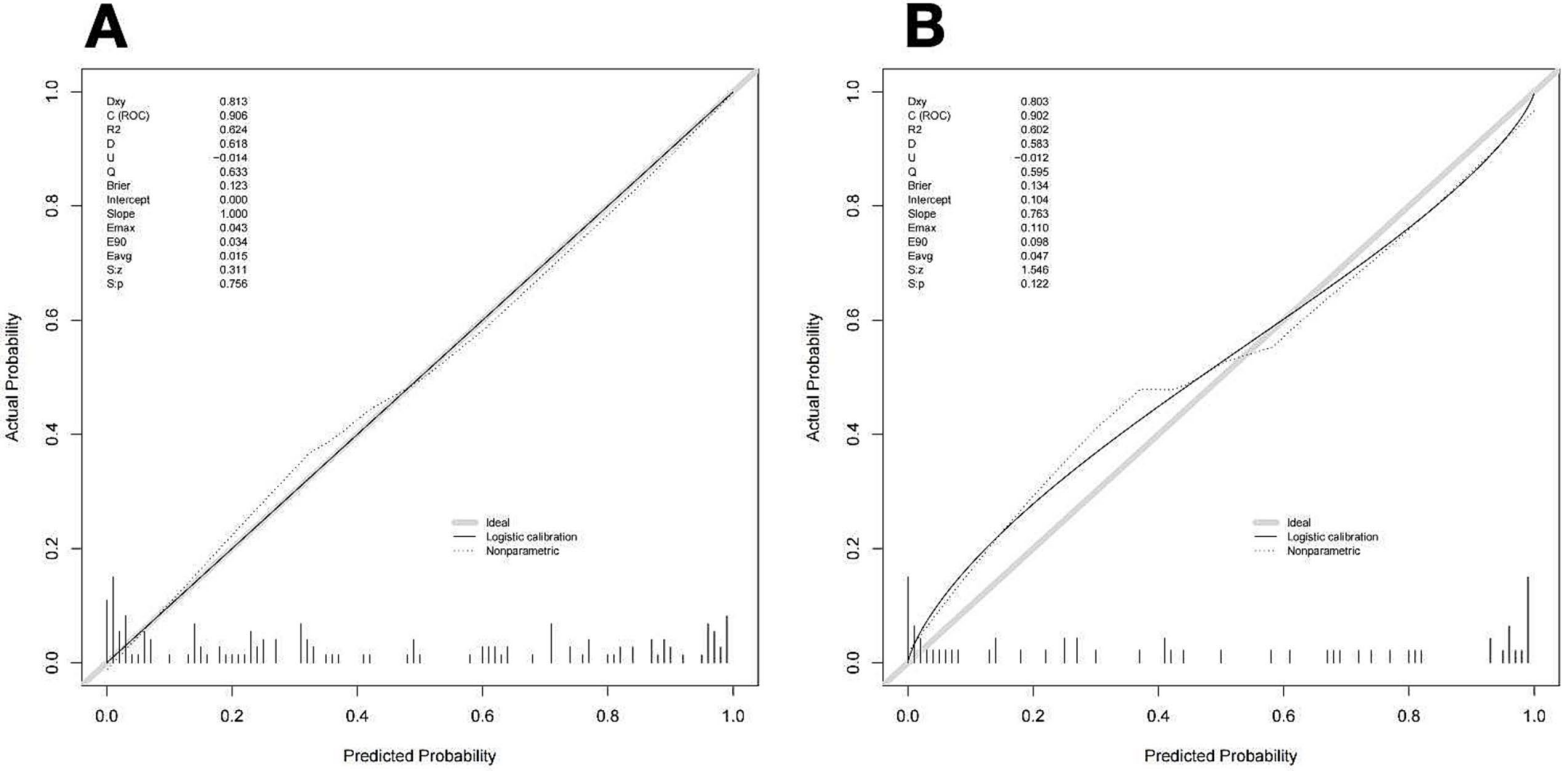
Calibration curve of the nomogram to predict vessel recanalization for the training cohort **(A)** and Validation cohort **(B)**. The proximity of a solid line (performance nomogram) to a dotted line (ideal model) directly correlates with the accuracy of the nomogram’s prediction.

One thousand bootstrap studies were used to authenticate and calibrate the nomogram. The calibration plots of the nomogram in the various cohorts are shown in Figure 6, showing a strong connection between the observed and anticipated recanalization. The findings indicated that the original nomogram remained applicable for the validation sets, and the calibration curve of this model closely approximated the ideal curve. This implies that the anticipated outcomes were in line with the actual observations.

**Figure 7:**
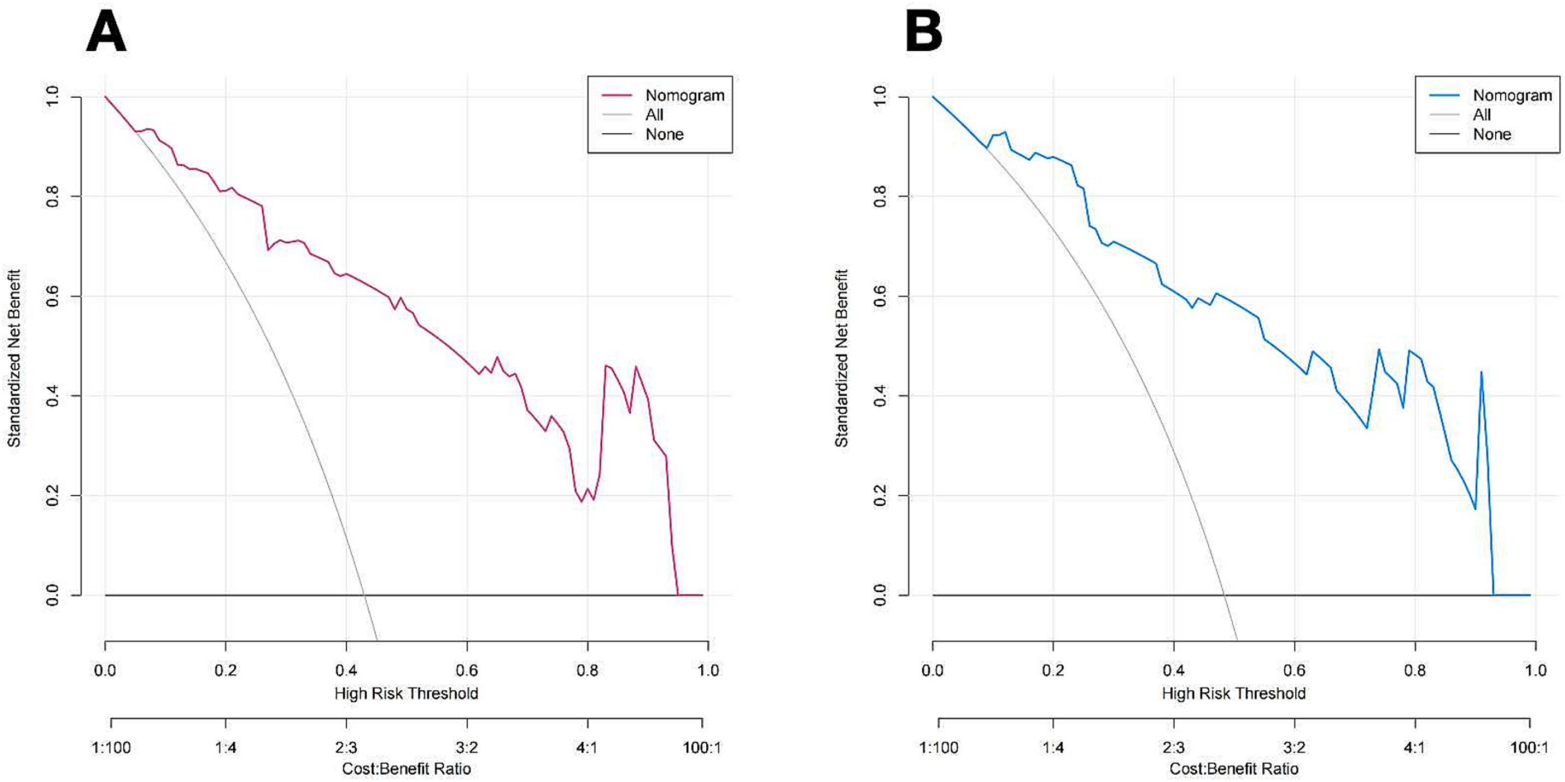
DCA of the nomogram of the training cohort **(A)** and Validation cohort **(B)**. The color line (red and blue) represents the prediction model, the grey line represents all patients with vessel recanalization, and the solid black line represents no patients with vessel recanalization. The nomogram offers substantial net benefits for clinical application.

**Figure 8:**
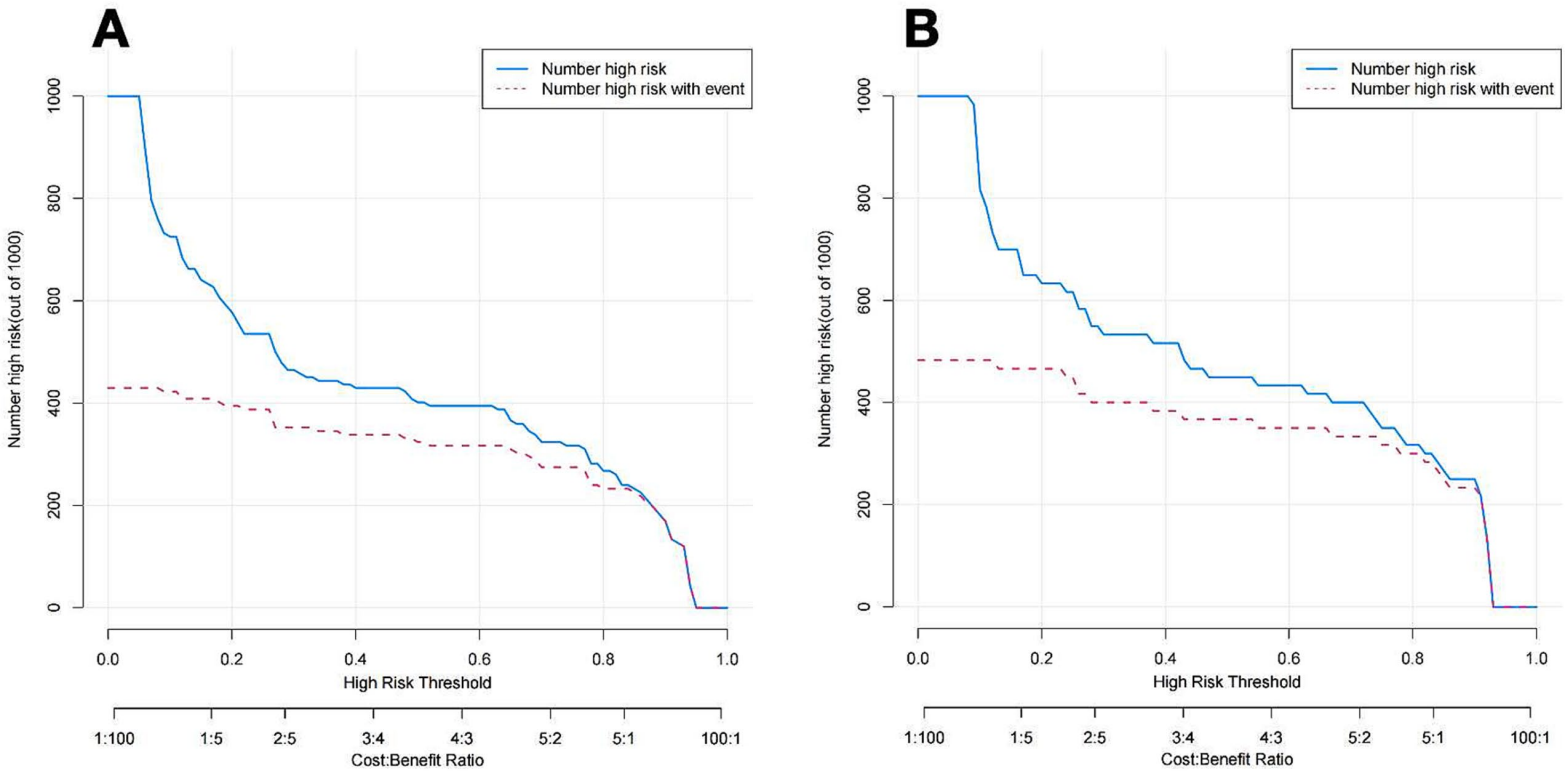
CIC of the nomogram of the training cohort **(A)** and Validation cohort **(B)**. The blue line represents the number of people judged to be at high risk by the model under different probability thresholds; the red lines shows the number of true positives for each threshold probability.

Figure 7 illustrates the DCA curves associated with the nomogram. The findings indicated that the nomogram exhibited a greater net advantage in the validation set when the threshold probability ranged from 10 to 75%. CIC analysis showed the clinical efficacy of the predictive model. When the threshold probability is greater than 80% of the prediction score probability value, the prediction model determines that the population of recanalization is highly matched with the actual population, which confirms the high clinical effectiveness of the prediction model (Figure 8).

### Risk stratification based on nomogram scores

Using the cutoff value of 0.5, 64.8% (92/142) of patients in the training cohort and 61.7% (37/60) in the validation cohort were classified as high-risk for non-recanalization. High-risk patients exhibited significantly lower recanalization rates (training: 12.0% vs. 88.0%; validation: 16.2% vs. 83.8%; *p* < 0.001) compared to low-risk patients, confirming the clinical utility of the nomogram in risk stratification.

## Discussion

In the current paper, we created a nomogram that utilizes CDU features and clinical parameters for forecasting vessel recanalization in EICAD patients. This nomogram was verified by analyzing ROC, calibration, DCA curves, and CIC for both the training set and validation set. The outcomes manifested in the nomogram may represent a useful tool for predicting vessel recanalization. Baracchini et al. analyzed 103 ICAD and VAD patients and concluded that the recanalization rates were 55.0% (33/60) and 46.5% (20/43), respectively (5). The prognosis of patients with CAD is strongly influenced by vessel recanalization, and clarifying the influencing factors of recanalization helps predict the recanalization rate and evaluate the prognosis of patients. Our findings underscore the clinical relevance of vessel recanalization in EICAD. The significantly lower incidence of cerebrovascular events in patients with recanalization (3.3% vs. 11.6%) suggests that successful recanalization may mitigate long-term thromboembolic risk. This aligns with prior studies reporting that persistent arterial occlusion in CAD is associated with higher stroke recurrence rates (16).

Some studies have used ultrasound to follow up patients with cervical artery dissection to explore the related factors of recanalization. Strunk et al. analyzed 183 dissected vessels and found that the number of dissecting vessels and the degree of lumen stenosis were independent risk factors (17). However, few studies have been reported to predict EICAD vessel recanalization by combining CDU features and clinical characteristics. Our results found that age, intramural hematoma, Intraluminal thrombus, and lumen stenosis degree were predictors of vessel recanalization. LASSO regression analysis can solve a variety of confounding variables as well as multicollinearity problems, thus providing a more accurate result. In addition, the LASSO can also filter the variables, making the model relatively simple and stable. The primary distinction between our work and other studies using logistic regression analysis is that we used LASSO regression analysis to screen the variables and combined traditional logistic regression analysis to obtain a more comprehensive model, thereby improving the prediction efficacy.

A previous study of 110 patients with vertebral artery dissections reported that younger patients had better clinical outcomes than older patients (18), which is consistent with our results. Our results showed that age was negatively correlated with vessel recanalization. Results from the CADISS trial also showed a significant association between increasing age and residual lumen stenosis and occlusion (19). The reasons may be as follows: the absorption of hematoma in CAD depends on fibrinolysis, while the function of the human fibrinolytic system is related to age, with the strongest effect in youth, and its function gradually decreases with the growth of age (20, 21). Some studies have reported that with the increase in age, the decrease of male androgen and female estrogen levels can lead to a decrease in fibrinolytic system activity (22, 23). Therefore, in older patients, the function of the fibrinolytic system is relatively reduced, with the dissolution and absorption of the intramural hematoma weakened, which eventually leads to the failure of vessel recanalization.

Some studies have reported that smoking may lead to CAD. Our study showed that smoking is negatively correlated with vascular recanalization, and the probability of vascular recanalization in smokers is significantly lower than that in non-smokers. The reason maybe that smoking can cause vascular smooth muscle proliferation, and vascular smooth muscle proliferation plays an important role in the occurrence, development and prognosis of vascular diseases (24, 25). Smoking will cause vasculitic changes, resulting in changes in the structure of blood vessels, which will affect the adhesion of the inner artery of the dissection blood vessels during the recovery process, and thus hinder the recanalization of the dissection blood vessels (24, 25).

Vicenzini et al. (26) conducted performed a 4-year ultrasound follow-up study on 14 CAD patients and found that degenerative intramural hematoma seemed to be associated with persistent occlusion and partial recanalization. The presence of degenerated hematoma in the carotid artery (CA) indicates that the occlusion can be uneven and recanalized, and the remodeling of intramural hematoma substances can sometimes take up to two years. Our findings showed that IMH may be a risk factor for vessel recanalization. The reason may be that when the hematoma changes to the chronic stage, the echo-intensity is gradually enhanced. The enhanced echo-intensity of the intramural hematoma represents the absorption of hemoglobin, as well as fibrosis. At this time, the vascular wall remodeling and the probability of vascular recanalization will be greatly reduced. CDU surveillance is beneficial to the early detection the echo-intensity of IMH, and patients can be treated in time, which is of great significance to improving the vessel recanalization rate.

Our findings showed that the recanalization rate of occlusion was 33.0%, and MVLR analysis indicated that vascular occlusion was an autonomous risk factor for complete recanalization. These outcomes corroborate the prior research conducted on CAD patients; the recanalization rate of stenosis is 46 to 90% and about 33 to 50% in patients with occlusion (27). As the lumen of the internal carotid artery is thick, the blood flow is high, and the blood pressure on the wall is large. When the intima of the artery is torn, the blood flow enters the false lumen under the action of high pressure, resulting in more serious intima tear and larger intramural hematoma formation, which is easy to cause severe stenosis or occlusion of the internal carotid artery lumen. Traenka et al. examined a total of 2,148 patients from many centers and reached a conclusion that dissection artery occlusion predicted a poorer prognosis and a complete recanalization rate in CAD patients (28). The reason may be related to the effect of arterial occlusion on vascular remodeling in CAD patients. The remodeling mechanism of arterial vessels is related to the shear force of the vascular wall. When CAD only causes arterial stenosis, the decrease in vessel diameter leads to an increase in vascular wall shear force, and the increased shear force can activate nitric oxide-dependent vasodilation, inhibit endothelial cell proliferation and inflammatory response, stabilize vascular endothelial cells, and enlarge the inner diameter of the artery cavity, then promote CAD vascular cavity complete recanalization (29–31).

The nomogram we have created has several clinical implications. Initially, based on the information available to us, this is the first nomogram to serve as a non-invasive tool based on CDU and clinical characteristics to predict vessel recanalization in patients with EICAD, aiding in better risk stratification. Moreover, the nomogram’s high-risk threshold (probability <50%) identifies patients with a low likelihood of spontaneous recanalization, who may benefit from intensified monitoring or early endovascular intervention. Future trials could validate whether targeting high-risk patients with adjunct therapies improves outcomes.

There are many constraints in our research that need to be recognized. Initially, the cohort consisted of patients only from a single facility, and the sample size was rather limited, perhaps limiting its representativeness to the broader community. Furthermore, the nomogram underwent validation in people from the same institution that produced the training cohort; external validation in diverse populations will be essential to confirm the generalizability of our findings. Future research should aim to validate our nomogram externally in different populations and settings. Additionally, integrating novel predictors or biomarkers could enhance the predictive accuracy of the nomogram, warranting further investigation. Lastly, treatment regimens (e.g., duration, phase-based adjustments, and compliance) were not systematically recorded in this retrospective study, which may have influenced recanalization outcomes. Future studies should prospectively control for therapeutic variables.

## Conclusion

We constructed a user-friendly and non-invasive nomogram based on CDU and clinical features to predict vessel recanalization in EICAD patients, which may facilitate early clinical evaluation to select individual therapies and thus improve patient outcomes.

## Data Availability

The raw data supporting the conclusions of this article will be made available by the authors, without undue reservation.
